# Use of Postpartum Care: Predictors and Barriers

**DOI:** 10.1155/2014/530769

**Published:** 2014-02-20

**Authors:** Jessica N. DiBari, Stella M. Yu, Shin M. Chao, Michael C. Lu

**Affiliations:** ^1^Health Resources Services Administration, Maternal and Child Health Bureau, Office of Epidemiology and Research, Division of Research, 5600 Fishers Lane, Room 18A-55, Rockville, MD 20857, USA; ^2^Los Angeles County Department of Public Health, Maternal, Child, and Adolescent Health Programs, Research Evaluation and Planning Division, 600 S. Commonwealth Avenue, 8th Floor, Los Angeles, CA 90005, USA

## Abstract

This study aimed to identify actual and perceived barriers to postpartum care among a probability sample of women who gave birth in Los Angeles County, California in 2007. Survey data from the 2007 Los Angeles Mommy and Baby (LAMB) study (*N* = 4,075) were used to identify predictors and barriers to postpartum care use. The LAMB study was a cross-sectional, population-based study that examined maternal and child health outcomes during the preconception, prenatal, and postpartum periods. Multivariable analyses identified low income, being separated/divorced and never married, trying hard to get pregnant or trying to prevent pregnancy, Medi-Cal insurance holders, and lack of prenatal care to be risk factors of postpartum care nonuse, while Hispanic ethnicity was protective. The most commonly reported barriers to postpartum care use were feeling fine, being too busy with the baby, having other things going on, and a lack of need. Findings from this study can inform the development of interventions targeting subgroups at risk for not obtaining postpartum care. Community education and improved access to care can further increase the acceptability of postpartum visits and contribute to improvements in women's health. Postpartum care can serve as a gateway to engage underserved populations in the continuum of women's health care.

## 1. Introduction

The postpartum care (PPC) visit is an important opportunity to assess the physical and psychosocial health of the mother [1]. The American Academy of Pediatrics (AAP) and the American College of Obstetricians and Gynecologists (ACOG) recommend that women, regardless of age, seek postpartum care between 4 and 6 weeks after childbirth [2]. The postpartum care visit may be utilized to counsel mothers on infant care and family planning, encourage breastfeeding, identify and treat medical conditions common to the postpartum period, and manage preexisting or emerging chronic conditions [2]. Despite the known benefits of the PPC visit, there are many access and utilization barriers to care [3]. As a result, Healthy People 2020 aims to increase the proportion of women, across demographic and socioeconomic boundaries, who attend a PPC visit after giving birth, thereby highlighting postpartum care as a national priority to promote the health of women and children [4].

Data on the utilization, content, and quality of the PPC visit are limited and often lack a comprehensive preventive care component [5]. According to the Centers for Disease Control and Prevention's (CDC) Pregnancy Risk Assessment Monitoring System (PRAMS) 2004, based on data from 11 states and New York City, 89% of US women who deliver attend a PPC visit [2]. However, the prevalence of women who attend a postpartum care visit is lower among certain subgroups. Approximately 71.2% of women who had received late prenatal care and 71% of women with ≤8 years of education attend a postpartum care visit. In addition, only 66% of women who did not receive prenatal care and 59.5% of women whose infants did not have newborn checkups obtain PPC [2]. Among the women who do attend the PPC visit, the content and quality of the care can vary substantially [6].

Many factors influence the decision to seek PPC. A study by Kogan et al. reported that the strongest indicator of whether a woman would obtain PPC was her use of prenatal care [7]. Other studies have reported that women with inadequate or no prenatal care are less likely to adhere to recommendations for continuity of care after birth, which include newborn visits, childhood immunizations, and postpartum visits [8]. Among a nationally represented sample of women who had a live birth in the 1988 National Maternal and Infant Health Survey (*N* = 9953), women who had no prenatal care had a 3.39 greater odds of not receiving PPC compared to women who had prenatal care [5]. A commitment to preventive health during pregnancy (i.e., prenatal care) may be a strong indicator of maternal health care utilization after birth.

Maternal physical and mental health status may also predict PPC utilization. New mothers are often sleep deprived, overwhelmed, and have limited time to tend to their personal health care needs. The intent of the pregnancy may also influence maternal regard for postpartum health [9]. Women with unintended pregnancies may find it difficult to come to terms with their pregnancy. After birth, postpartum depression and other mental health conditions may result, impacting the woman's willingness to take initiative and follow through with medical appointments [10]. Alcohol and drug use can further hinder a woman's ability to attend to her health care needs. In addition, women who had a baby born preterm or low birth weight may be less likely to seek PPC, as their attention and time are devoted to the care of the newborn [5, 11]. Furthermore, enduring traumatic events, such as a still birth or infant death have a major impact on mental health status, which may result in low PPC utilization rates [7].

Sociodemographic factors play an important role in PPC utilization as well. A number of subpopulations are less likely to seek PPC. Some studies report that women with less than a high school education, less than 26 years of age, a household income of less than $20,000, and high parity are associated with a low acceptance rate of the PPC visit [2, 5, 7]. The disparities in PPC utilization require attention to improve access to quality care for women across socioeconomic and demographic boundaries.

Several studies have analyzed barriers to PPC utilization. Access barriers further decrease the likelihood that a new mother will schedule a PPC visit. Medicaid programs serve a higher proportion of pregnant women of low socioeconomic status with special needs than do private insurers [12]. Although Medicaid recipients receive hospital coverage, after birth outpatient visits such as PPC visits, are not always covered expenses [13]. As a result, these women tend to seek acute, reactive care and do not have a long-term health care plan. Recent legal immigrants experience additional barriers to care as they are ineligible for Medicaid services and language barriers can be a deterrent to seeking care as well [14]. PPC among this subgroup is often limited to emergency care services. Lack of comprehensive health insurance coverage is a considerable challenge to meeting maternal healthcare needs. Furthermore, cultural and religious beliefs influence an individual's regard for the postpartum visit. Social support, including partner involvement, can have a bearing on perceived feasibility of scheduling a PPC visit while caring for a newborn. Logistical barriers such as inaccessible transportation, long waits during appointments, and lack of child care further limit the likelihood of a postpartum visit [3].

In light of the expanding Hispanic population in the US, our study is focused on a predominantly Hispanic multiethnic sample of low-income women. To address gaps in the literature, our study used data from a recent probability sample of mothers from Los Angeles County to study the determinants of and barriers to PPC utilization. To our knowledge, this is the first study to date to highlight actual and perceived barriers to PPC in this subpopulation.

## 2. Materials and Methods

Data from the 2007 Los Angeles Mommy and Baby (LAMB) study (*N* = 4, 075) were used to examine the extent to which postpartum care utilization is related to sociodemographic characteristics and to identify factors that reduce the likelihood of postpartum care utilization among women living in Los Angeles (LA), California. The LAMB study was a cross-sectional, population-based study that examined maternal and child health outcomes during the preconception, prenatal, and postpartum periods. Eligible participants were LA residents who had a live-birth in LA County in 2007.

This study was a collaborative effort between the University of California, Los Angeles (UCLA) and the Los Angeles County Department of Maternal, Child, and Adolescent Health. The sample was derived from a stratified random sample of census tract defined neighborhoods. A total of 10,000 surveys were mailed to eligible women within 6 months after delivery. The response rate was 56%, after adjusting for faulty addresses, language issues, maternal deaths, and loss of follow-up due to inability to locate the respondent. The recruitment process consisted of a (1) notification letter 4 months after child birth, (2) mailed questionnaires, (3) mailed reminder letters, (4) and telephone follow-up to nonresponders. The questionnaires were translated into Spanish and Chinese and a telephone translation service provided access in 88 languages. Information about the recruitment strategy and methodology has been published in detail elsewhere [15]. Data from completed surveys were linked to birth certificate data resulting in a final analytic sample of 4,075.

This study was approved by both the Los Angeles County Department of Public Health and the UCLA Institutional Review Boards (IRBs) in 2007.

### 2.1. Variables

The current analysis focused on the determinants of, and barriers to, postpartum care. Respondents were asked whether or not they obtained a postpartum care visit (yes/no). A range of maternal sociodemographic factors including race (Non-Hispanic White, Hispanic, Non-Hispanic Black, Asian/Pacific Islander (API), and Native American), age in years (0–16, 17-18, 19–29, 30–39, 40–49), marital status (married, separated/divorced, widowed, never married but living together, never married but living apart), income (<$20,000, $20,000–39,000, $40,000–59,000, $60,000–99,000, >$100,000), and education (less than high school, high school graduate, some college or more, and not stated) were included in the analyses (Table 1). Race/ethnicity data were obtained from the California birth certificates. When two races/ethnicities were provided, the first listed race/ethnicity was used.

Additional variables of interest included insurance type (no insurance, medi-Cal/medicaid, private insurance, other), prenatal care utilization (yes/no), care received prior to pregnancy (reasons for seeking care: to have a healthy pregnancy, chronic medical problem, problem with previous pregnancy, expected to get pregnant, encouraged by doctor or nurse), intendedness of pregnancy (yes, yes but not trying very hard, trying hard to keep from getting pregnant, or neither trying nor preventing pregnancy), preterm birth, low birth weight (yes/no), and whether or not the infant attended a newborn visit (yes/no).

### 2.2. Statistical Analyses

We analyzed the data using SAS 9.3 (SAS Institute Inc., Cary, NC). Weights were added to account for the sampling design and survey nonresponse. To test differences between women who did and did not obtain postpartum care on select socio-demographic factors and other possible determinants, *χ* ^2^ analyses were performed (Table 2). Multivariate logistic regression analyses were used to identify predictors of postpartum care utilization adjusting for selected covariates (Table 3). Adjusted odds ratios (ORs) and 95% confidence intervals (CIs) were computed using the beta coefficients (*β*) and standard errors (SEs) from the multivariable logistic analyses.

## 3. Results

The final analytic sample consisted of 4,075 women in LA County. The sample was predominantly Hispanic, between 19 and 39 years old, married, had an annual income <$20,000, and obtained a high school-level education or less. Table 2 displays the bivariate analyses of select maternal demographic and socioeconomic characteristics by postpartum visit status. In the bivariate analyses, maternal race/ethnicity, age, income, marital status, education, pregnancy intendedness, prenatal care, care received prior to pregnancy, and preterm birth/low birth weight were all significantly associated with PPC use.

Table 3 shows results of the multivariable analysis on the association between predictors and the lack of postpartum care utilization while controlling for confounders. In Model 1, Hispanic mothers were significantly less likely to lack a PPC visit compared with non-Hispanic White mothers (OR = 0.57, 95% CI 0.38, 0.87). Mothers aged 30 to 39 (OR = 0.68, 95% CI 0.51, 0.91) were less likely to lack a PPC visit compared to mothers aged 19 to 29. Mothers from families with income less than $20,000 were almost three times as likely to lack a PPC visit (OR = 2.89, 95% CI 1.43, 5.82) compared to mothers with households of >$100,000. Separated or divorced and never married mothers were more likely to lack a PPC visit compared to married mothers. Mothers who reported not trying hard to get pregnant or trying to prevent pregnancy were also more likely to lack a PPC visit, compared to those who were trying. Mothers who did not attend prenatal care were more than 3 times as likely to lack a PPC visit (OR = 3.08, 95% CI 1.68, 5.63). When insurance status replaced family income in Model 2, mothers who had Medi-Cal insurance were twice as likely to lack a PPC visit compared to those on private insurance (OR = 2.19, 95% CI 1.54, 3.11). Hispanic ethnicity, maternal age, marital status, pregnancy intendedness, and prenatal care utilization remained significant predictors.

**Figure 1:**
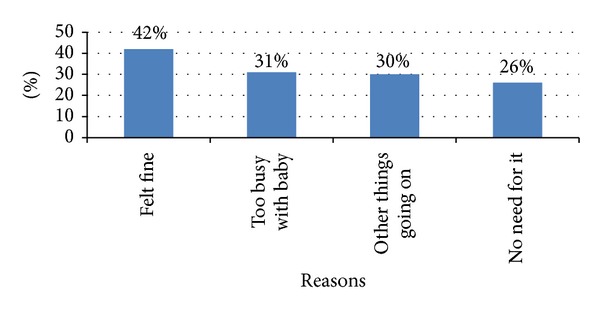
Top reasons reported for not receiving a postpartum checkup (*N* = 327).

Figure 1 shows the most commonly reported reasons for not receiving a postpartum check-up. Women reported that they felt fine, were too busy with the baby, had other things going on, and felt there was no need for PPC.

## 4. Discussion

Our paper highlights the many factors influencing a woman's decision to seek postpartum care. Our study population was multiethnic, predominantly Hispanic and low-income, based on the demographics of Los Angeles County. In the multivariable analyses, postpartum care utilization was lowest among women who were low income, separated/divorced, and had an unplanned pregnancy. In our study, Hispanic women were more likely to obtain PPC compared to non-Hispanic White women. Consistent with other studies, prenatal care was the strongest predictor of postpartum care utilization [5, 7, 8]. Women trying to get pregnant may be more inclined to seek care prior to, during, and after pregnancy. One would expect that a continuum of maternal care may reflect a women's commitment to life-long health. However, on the contrary to our expectations, care received prior to pregnancy was not a significant determinant of PPC utilization. In addition, obtaining a newborn visit was independent of the PPC visit. The perinatal period represents a window of opportunity to engage and educate women about the importance of postpartum care.

Results from this study indicate that targeted educational efforts are needed. The top 5 reasons for not seeking PPC indicate that women who lacked PPC did not consider the PPC visit a high priority (Figure 1). Interestingly, access to care was not perceived as a top reason for not obtaining PPC. Interventions aimed at changing attitudes and perceptions about the importance of the PPC visit must include individuals, families, and communities. The Hispanic community, where familial support is of high value [16], had the highest adjusted rates for postpartum care use. A multitiered approach, targeting individuals, families, and communities may be necessary to improve PPC utilization rates. Women who do not have an adequate support system in place may find it exceedingly difficult to set aside time to care for their personal healthcare needs. For the purpose of this analysis, marital status was viewed as a proxy for support. In addition to a supportive spouse, extended family support can also facilitate a woman's ability to seek PPC. Encouragement from one's spouse and/or family member(s) can provide the necessary impetus for scheduling a postpartum visit. Even gentle nudging from friends, neighbors, and respected individuals in the community can elicit the motivation necessary to seek care.

In addition to educational barriers, access barriers are evident as well. PPC must be made available to women who do not have private insurance. Medi-Cal is the Medicaid program available to residents in the State of California. This public health insurance program provides health care services to low-income individuals and families. In California, certain eligibility requirements apply to receive 60 days of PPC through Medi-Cal services. If the eligibility requirements are met, covered services include hospital and scheduled office visits during puerperium, assessment of uterine involution, and contraceptive counseling [17]. Comprehensive postpartum care including nutrition, psychosocial, or health education services is billed fee for service [17]. Furthermore, the postpartum care eligibility period does not cover conditions unrelated to the pregnancy, such as urinary tract infection, respiratory infection, hepatitis, preexisting hypertension, cholecystitis, appendicitis, abnormal pap smear, and cancer [17]. Although some postpartum services are available, eligibility restrictions likely limit the accessibility of postpartum care for select subgroups.

Our findings indicate that women enrolled in Medi-Cal are twice as likely to lack a PPC visit compared to those with private insurance. These findings further support the need for national and state level policies addressing barriers to postpartum care. The Affordable Care Act addresses this need through the inclusion of provisions to support pregnant and postpartum women [18]. Under the Affordable Care Act, maternity and newborn care is considered one of the ten essential health benefits [19]. Furthermore, the Affordable Care Act helps make preventive care affordable and accessible by requiring new health plans to cover and eliminate cost sharing for preventive services [18].

Several limitations must be considered when interpreting our findings. The LAMB study was a cross-sectional study based on a sampling of live birth certificates. It relied on maternal recall and may be subject to bias. In addition, the mother's current relationship with the baby's father may differ from their relationship status during pregnancy and as a result has a bearing on the survey responses. As this study took place in LA County, findings may not be generalizable. However, this study contributes to the growing body of the literature emphasizing the importance of the postpartum visit and identifying barriers to PPC.

## 5. Conclusions

In our study population, despite the many barriers to obtaining postpartum care, Hispanic women were more likely to receive postpartum services compared with non-Hispanic White mothers. Both financial resources and familial support appear to influence PPC utilization rates. An interesting finding of our study was that perceptions about the barriers to postpartum care were the result of a perceived low value of the postpartum visit. This indicates a need for health care facilities and providers to make concerted efforts to increase knowledge about the importance of the postpartum visit, enhance the use or design of medical encounters, identify community resources, and develop targeted interventions [3]. The prenatal care visit is one early opportunity to educate women about the importance of PPC. Providers can influence perceptions about PPC, increase its acceptability, and contribute to significant improvements in women's health.

## Figures and Tables

**Table 1:** Sociodemographic and maternal characteristics of study sample, 2007 LAMB (*N* = 4075).

	*N*	%	SE
Maternal race/ethnicity			
Non-Hispanic White	794	12.3	0.85
Hispanic	2495	74.1	1.30
Non-Hispanic Black	244	4.6	0.46
Asian/pacific islander	501	8.3	0.75
Native American	41	0.7	0.12
Maternal age, years			
0 to 16	44	1.5	0.23
17 to 18	176	6.0	0.45
19 to 29	1970	51.5	0.96
30 to 39	1716	37.8	0.95
40 to 49	169	3.3	0.29
Family income			
<$20,000	1509	44.7	1.22
$20,000–39,000	858	21.8	0.74
$40,000–59,000	402	8.2	0.48
$60,000–99,000	530	9.5	0.56
>$100,000	536	8.5	0.67
Not stated	240	7.3	0.51
Marital status			
Married	2373	52.7	1.08
Separated/divorced	103	2.9	0.31
Widowed	6	0.2	0.07
Never married but living together	1098	31.5	0.99
Never married but living apart	431	11.0	0.57
Not stated	64	1.7	0.22
Maternal education			
Less than HS	1019	37.4	1.32
HS Grad	1045	25.4	0.79
Some college or more	873	18.9	0.71
Not stated	1138	18.3	0.99
Pregnancy intendedness			
Yes	1181	27.7	0.79
Yes, but not trying hard	481	11.2	0.52
No, trying hard to keep from getting pregnant	611	14.7	0.62
Neither trying nor preventing pregnancy	1735	44.2	0.86
Not stated	67	2.1	0.29
Prenatal care			
Yes	4019	98.5	0.21
No	56	1.5	0.21
Care received prior to pregnancy			
Yes	1212	26.7	0.79
No	2184	56.0	0.89
Not stated	679	17.4	0.63
Preterm birth/low birth weight			
Yes	739	15.7	0.51
No	3336	84.3	0.51
Newborn visit			
Yes	3980	97.4	0.26
No	62	1.7	0.22
Not stated	33	0.9	0.16
Insurance status			
No insurance	1339	40.2	1.05
Medi-Cal	788	22.0	0.89
Private	1692	32.3	1.16
Other	119	2.3	0.22
Not stated	137	3.2	0.29
Postpartum care			
Yes	3748	91.7	0.47
No	327	8.3	0.47

**Table 2:** Maternal demographic and socioeconomic characteristics by postpartum visit status. 2007 LAMBS.

	*N*	Has postpartum visit		Lacks postpartum visit		Chi square *P* value
		3748 (92%)		327 (8%)	
		%	SE	%	SE
Maternal race/ethnicity						**0.0032**
Non-Hispanic White	794	92.7	1.11	7.3	1.11	
Hispanic	2495	91.7	0.54	8.3	0.54	
Non-Hispanic Black	244	85.4	2.27	14.6	2.27	
Asian/pacific islander	501	93.8	1.16	6.2	1.16	
Native American	41	90.2	4.65	9.8	4.65	
Maternal age, years						**<0.0001**
0 to 16	44	75.4	6.72	24.6	6.72	
17 to 18	176	87.7	2.75	12.3	2.75	
19 to 29	1970	90.2	0.68	9.8	0.68	
30 to 39	1716	94.7	0.56	5.3	0.56	
40 to 49	169	95.3	1.86	4.7	1.86	
Family income						**<0.0001**
<$20,000	1509	89.0	0.82	11.0	0.82	
$20,000–39,000	858	93.3	0.90	6.7	0.90	
$40,000–59,000	402	93.3	1.37	6.7	1.37	
$60,000–99,000	530	95.9	0.96	4.1	0.96	
>$100,000	536	97.3	0.80	2.7	0.80	
Not stated	240	90.1	1.97	9.9	1.97	
Marital status						**<0.0001**
Married	2373	94.9	0.49	5.1	0.49	
Separated/divorced	103	85.1	3.64	14.9	3.64	
Widowed	6	84.9	14.20	15.1	14.20	
Never married but living together	1098	89.2	0.90	10.8	0.90	
Never married but living apart	431	85.0	1.80	15.0	1.80	
Not stated	64	93.1	3.10	6.9	3.10	
Maternal education						**0.0004**
Less than HS	1019	90.5	0.97	9.5	0.97	
HS grad	1045	90.8	0.91	9.2	0.91	
Some college or more	873	91.4	0.91	8.6	0.91	
Not stated	1138	95.7	0.65	4.3	0.65	
Pregnancy intendedness						**<0.0001**
Yes	1181	95.3	0.67	4.7	0.67	
Yes, but not trying hard	481	91.4	1.41	8.7	1.41	
No, trying hard to keep from getting pregnant	611	89.5	1.36	10.5	1.36	
Neither trying nor preventing pregnancy	1735	90.2	0.76	9.8	0.76	
Not stated	67	92.6	3.17	7.4	3.17	
Prenatal care						**<0.0001**
Yes	4019	92.0	0.46	8.0	0.46	
No	56	73.6	6.12	26.4	6.12	
Care received prior to pregnancy						**0.0177**
Yes	1212	93.8	0.73	6.2	0.73	
No	2184	91.0	0.68	9.0	0.68	
Not stated	679	90.7	1.14	9.3	1.14	
Preterm birth/low birth weight						**0.0189**
Yes	739	89.5	1.13	10.5	1.13	
No	3336	92.1	0.50	7.9	0.50	
Newborn visit						0.09
Yes	3980	91.9	0.46	8.1	0.46	
No	62	85.1	4.62	14.9	4.62	
Not stated	33	85.4	6.90	14.6	6.90	
Insurance status						**<0.0001**
No insurance	1339	92.2	0.74	7.8	0.74	
Medi-Cal	788	85.6	1.27	14.4	1.27	
Private	1692	94.7	0.59	5.3	0.59	
Other	119	97.3	1.60	2.7	1.60	
Not stated	137	93.8	2.25	6.2	2.25	

Bolded text indicates the association was statistically significant.

**Table 3:** Adjusted odds of lacking a postpartum visit among women who gave birth in Los Angeles County. 2007 LAMBS.

	Model 1			Model 2		
	OR	95% CI		OR	95% CI	
Maternal race/ethnicity						
Non-Hispanic White	Reference			Reference		
Hispanic	**0.57**	**0.38**	**0.87**	**0.64**	**0.43**	**0.96**
Non-Hispanic Black	0.99	0.59	1.65	0.99	0.59	1.66
Asian/pacific islander	0.76	0.46	1.25	0.77	0.46	1.27
Native American	0.75	0.24	2.32	0.89	0.28	2.84
Maternal age, yrs						
0 to 16	2.11	0.97	4.60	1.95	0.85	4.44
17 to 18	0.95	0.56	1.63	0.92	0.54	1.55
19 to 29	Reference			Reference		
30 to 39	**0.68**	**0.51**	**0.91**	**0.66**	**0.49**	**0.88**
40 to 49	0.60	0.25	1.46	0.56	0.23	1.35
Family income						
<$20,000	**2.89**	**1.43**	**5.82**			
$20,000–39,000	2.06	0.98	4.30			
$40,000–59,000	2.05	0.94	4.45			
$60,000–99,000	1.49	0.66	3.35			
>$100,000	Reference					
Not stated	**2.63**	**1.14**	**6.04**			
Marital status						
Married	Reference			Reference		
Separated/divorced	**2.15**	**1.16**	**3.96**	**2.39**	**1.28**	**4.49**
Widowed	1.55	0.16	15.14	1.68	0.20	14.17
Never married but living together	**1.62**	**1.20**	**2.18**	**1.63**	**1.20**	**2.23**
Never married but living apart	**2.04**	**1.39**	**2.98**	**2.17**	**1.47**	**3.22**
Pregnancy intendedness						
Yes	Reference			Reference		
Yes, but not trying hard	**1.87**	**1.19**	**2.94**	**1.90**	**1.20**	**3.01**
No, trying hard to keep from getting pregnant	**1.64**	**1.05**	**2.57**	**1.62**	**1.04**	**2.54**
Neither trying nor preventing pregnancy	**1.59**	**1.09**	**2.31**	**1.58**	**1.09**	**2.30**
Prenatal care						
Yes	Reference			Reference		
No	**3.08**	**1.68**	**5.63**	**2.91**	**1.57**	**5.39**
Care received prior to pregnancy						
Yes	Reference			Reference		
No	1.09	0.78	1.52	1.20	0.86	1.68
Preterm birth/low birth weight						
Yes	1.28	0.97	1.69	1.30	0.98	1.72
No	Reference			Reference		
Newborn visit						
Yes	Reference			Reference		
No	1.52	0.73	3.17	1.55	0.73	3.26
Insurance status						
No insurance				1.24	0.87	1.77
Medi-Cal				**2.19 **	**1.54 **	**3.11 **
Private				Reference		
Other				0.47	0.14	1.62

OR: odds ratio; SE: standard error; CI: confidence interval.

Bolded text indicates the association was statistically significant.
